# Clinical Lecture on Acute Albuminuria

**Published:** 1871

**Authors:** Wm. Abram Love

**Affiliations:** Prof. Physiology, in Atlanta Medical College; Atlanta, Ga.


ATLANTA

### Medical and Surgical Journal.

NEW SERIES.

VOL. IXB JULY AND AUGUST, 1871. NOS. 4 & 5.

*Clinical Lecture on Acute Albuminuria.* By Dr. ABRAM LOVE, M.D., Prof. Physiology, in Atlanta Medical College.

[Reported by EDWARD P. INGRAHAM, M.D.]

ALBUMINURIA ACUTE.

*Gentlemen.*—The first case presented for our consideration, this morning, is one of peculiar interest, by reason of its nature and complications. Mrs. C. K., æt, 42, native, has been suffering for the past week with asthma, to attacks of which she has been subject from childhood. On the 21st (about a week ago), while suffering from nettle-rash, to obtain relief, she bathed herself in cold water, at the time being very warm and perspiring freely. During the ensuing night she slept very little, her breathing being difficult, and having pains in the back with heaviness about the head. The following day she found more or less swelling of the extremities and face, the back symptoms increased, urine scanty, no perspiration, and continued difficult breathing ; says she has been tired and exhausted, and getting worse ever since she took the bath.

On examination, you notice that there is not only much œdema with difficult pitting on pressure of the lower extremities, but of the upper, particularly the left, and more or less of the general surface, especially of the chest, neck and face.

There is in addition, an effusion in the peritoneal cavity, and the same, to some extent, in the pleural, both serving to obstruct respiration, in addition to the asthma. The action of the heart is labored, otherwise normal. She passes, as near as can be ascertained, not more than six or eight ounces of urine in twenty-four hours ; though micturition is frequent, the quantity voided is small. A specimen of the urine we have here for examination. You see that it is pale, almost colorless. Specific gravity, as indicated by the urinometer, 1015. On boiling it you observe there is a white precipitate, (of about one eighth by volume.) This precipitate which is not dissolved, on the addition of an excess of nitric acid, is *coagulated albumen.* Another test for albumen which we show you, is a mixture of equal volumes of carbolic and acetic acids. A few drops of this added to the suspected liquid throws down a white precipitate if albumen exists. This test was recently recommended by Meynott Tidy. Aside from its being very reliable, it is more convenient than heat and nitric acid.

Dr. Ingraham will exhibit to you a specimen of the urine under the microscope ; in this you will see quite a number of tube-casts, granular, and more or less broken up, varying in size, though the majority are quite large, much larger than are usually seen. Also in the field you will observe a number of kidney epithelia and blood corpuscles.

We have then, gentlemen, in this case, a combination of diseases ; that is to say, we have dropsical effusions in the cellular tissues that might be denominated anasarca—of the pleural cavity, that might be diagnosed hydrothorax—in the peritoneal cavity, ascites. The character of the respiration and emphysematous condition of the lungs indicate asthma. The chemical tests and microscopical examination of the urine, prove unmistakably that we have a case of *acute albuminuria,* or a form of Bright's disease of the kidney. It is to this last that we will direct our special attention, as it is upon a correct understanding of the pathological conditions existing, that our success in treatment must depend.

The attention of the profession was called to this disease first by Bright, of London, in 1827, since which time it has borne his name. We meet with the disease existing in two distinct forms, as acute and chronic, and have also the acute supervening on the chronic.

The *symptoms of acute albuminuria* resemble ordinary inflammation of the kidney in the frequent desire to void urine.

Micturation painful, sometimes difficult, in many cases passing in drops, diminished in quantity, containing albumen, in some instances almost to a gelatinous consistency. The urine froths on shaking (as this does), is clear, may be colored amber or varying in shades to a blood-red, even containing blood. It is sometimes turbid, from containing oily matter or mucous.

The albumen in some cases amounting to as much as 25 to 30 parts per thousand by weight, but is generally about 10 to 20. By aid of the microscope, which is invaluable in all diseases where the kidneys are involved, we find floating in the urine tube-casts and epithelia, or cylinders of fibrin, or blood, or epithelial desquamation, moulded into shape by the tubuli uriniferi and thrown out with the kidney excretion. The specific gravity is generally normal, or a little below, the salines always diminished in quantity.

Where the *chronic form supervenes* on the *acute*, we have but a complicated continuation of less violent symptoms, resulting in serous effusions in the tissues with organic degeneration of the kidneys, and the concomitants of a broken down constitution.

In that form of the disease generally denominated *chronic*, its incursion is much more stealthy, and the symptoms very much obscured ; in this form, too, months and even years may elapse before much effect is produced on the general health. When it begins to affect the constitution, the patient grows gradually weak, digestion becomes deranged, has frequent desire to micturate, with coming and going pain in the lumbar region, particularly in the region of the kidneys. The quantity of urine diminishes, but as no special emergency seems to demand medical aid, little or no attention is given to the symptoms until the face, extremities or some other or all of the surface of the body becomes swollen, when dropsy is suspected, and the physican consulted. At this stage we find, in addition to the œdema, generally, though not always, tenderness of the kidneys and the quantity as well as the density of the urine, diminished ; the skin presents a dry, bloodless appearance, gastro-intestinal disturbances indicated by nausea or vomiting, thirst inordinate, a feeling of languor, lassitude and drowsiness. The dryness and dusky hue of the surface increases almost to a purple, especially along the dorsal region, where tactile sense is obtunded. At this stage, slight exposure to cold brings up an acute attack, fever ensues and all the symptoms presented in the acute form may follow and subside again, leaving the patient suffering from the previously existing chronic disease. In this condition the patient is particularly susceptible to the incursion of other diseases of an inflammatory nature, acute or chronic, especially of the viscera, as of pneumonia, pleurisy, pericarditis, bronchitis, violent and fatal dysentery, exhaustive diarrhœa or organic disease of the heart, liver and spleen, or rapidly fatal peritonitis. Again, the patient may pass off with coma or appoplexy, as is the tendency also in the acute form of the disease.

As we shall see in the pathology, the *cause* of this disease may be set down as *suppressed cutaneous secretion during obstructed respiration.* Enumerated among the leading causes, are pressure on the diaphragm by a gravid uterus or abdominal tumours, phthisis pulmonalis, asthma (as in this case), œdema glottis, congestion, and other diseases obstructing or interfering with the proper arterialization of the blood. The peculiar state of the blood in scarlet fever, measles, pyæmia or septicæmia, or the passive congestions of typhoid diseases, are also regarded as causes. Changes in the blood from breathing an atmosphere deficient in oxygen, or containing oxygen more or less devitalized from want of proper circulation in the proper atmospheric gasses, or from being deprived of its ozone or atmospheric electricity by the affinity of the ozone for such agents as attract and appropriate it. This we find occurring in the vicinity of decaying walls of houses, the feathers of feather beds on which some asthmatics can not sleep, or in some localities where substances are undergoing oxydation at the expense of the ozone of the atmosphere. Where any of these conditions exist and a suppressed cutaneous secretion supervenes, the kidneys are made to bear the burden, which, if continued or exaggerated, bring up the phenomena of Bright's disease.

Then, when we inquire into the pathology of the disease, we find that it arises in the double effects following suppression of the cutaneous functions and obstructed respiration. These effects are,

I. The general disturbed respiration interfering with the proper arterialization of the blood, results in a greater or less degree in the non-oxydation of the nitrogenous materials or albumenoids of the liquor sanguinis; as a consequence of this, the production of urea is diminished on the one hand, while albumen is eliminated on the other. The sulphates are also diminished in albuminuria, from the non-combustion of the albumenoids, to which, reference has just been made.

II. In health, about one-half of the fluids are eliminated through the cutaneous surface, whilst the other half is passed off as a kidney excretion. The sudden suppression of cutaneous action entails a double duty upon the kidneys. The non-oxydation of the nitrogenous material leaves in the blood, albumen not appropriated, hence it must be forced out through the thin walls of the kidney capillaries—the more fluid portions that should have been passed off through cutaneous exhalents, being effused into the cellular tissues or into the serous cavities, as we find in the case before us—a general œdematous condition of the surface of the body, together with hydrothorax and ascites.

III. These mechanical obstructions—albumen in the blood and checked cutaneous action — create a congested condition of the peripheral circulation in the capillaries, resulting in excessive engorgement of the thin-walled vessels of the kidneys, and the diminution of the quantity of urine excreted. Hence, by increased irritation from the forced elimination of albumen, there is set up organic disease in the tubules, at the same time desquamation of their epithelia and the discharge of tube-casts, as exhibited to you under the microscope. Another sequence of these mechanical obstructions is an increased pressure on the heart, producing' if long continued, dilation or hypertrophy of the ventricles. At the same time, the system suffers serious changes in nutrition, from want of assimilation of the food elements or albumenoids, that should have been consumed for growth and repair of tissue; this, together with the non-elimination of effete matter, will readily account for the symptoms of unrest and general disturbance of the brain and nervous system. Should the nitrogenous elements existing in the blood as albumen undergo rapid combustion, resulting in the production of urea during the suppression of cutaneous and kidney excretion, we must expect to find, as the legitimate result, symptoms of uræmia terminating in fatal coma.

In the treatment, gentlemen, we must be governed in our use of therapeutic agents by the pathological conditions existing. The first thing, then, to be accomplished is to re-establish cutaneous action, and to do this we have no better means than the vapor bath, which can be obtained, in the absence of more convenient apparatus, by pouring water on hot bricks and covering the body with blankets, allowing the vapor from the bricks to pass beneath the covering until the skin is relaxed and free perspiration induced, or the same object may be accomplished by the use of tepid saline baths.

The next indication to be fulfilled is to remove the obstructions to respiration, or produce the proper arterialization of the blood. For this purpose the inhalation of pure oxygen gas, did we have it at command, would seem to be specially indicated. In the absence of this, we must resort to other means to accomplish the same end; that is, relieve the spasmodic constriction of the air passages, and thereby admit a greater amount of pure air. With this idea, we shall administer to this patient *chloroform by the stomach,* combined with tr. opii camph. as a nerve stimulant and expectorant. Another indication to fulfill is to aid the heart by cardiac stimulants, to overcome the obstruction in the capillaries. For this purpose we use digitalis, which fills a double indication of strengthening the heart's action and promoting the kidney excretion.

While we are directing our attention to the accomplishment of these objects, we must also take into consideration the vitiated condition of the blood—give tone to its corpuscles—at the same time guarding against the rapid production of urea, to avoid which we must shun all agents tending to the rapid oxydation of the nitrogenous elements or albumenoids existing in the liquor sanguinis, until we have re-established the secretions. We therefore resort to the use of tr. ferri chlor. to build up the red corpuscles, and by the vital chemical action and stimulating effects on the nervous system, of the chlorine, and in the assimilation and elimination of the albumenoids, without producing urea too rapidly. Together with these we give iodine (if the stomach will bear it), in the form of potas. iod., to promote absorption. When the normal respiration and cutaneous action have been fairly established, through the use of these remedies, we may aid in the removal of the effusion, by the diuretic and hydragogue action of potas. bitart., freely diluted and given *ad lib.*

We therefore order for our patient — first, vapor baths twice a day; secondly, chloroform fʒss., and tr. opii camph. fʒiss. every two or three hours, until the asthmatic symptoms are overcome, and then as often as occasion may require to keep them under control; thirdly, tr. ferri. chlor. gtt. xxv., tr. digitalis gtt. x., potas. iod. gr. v., three times a day in water.

---

[NOTE.—*June 30th.*—Cutaneous action thoroughly re-established; the œdem rapidly disappearing; respiration free and easy; the quantity of urine began to increase soon after commencing treatment, and has gradually increased to now more than normal; the patient is every way more comfortable. In consequence of the iodide of potassium disagreeing with the stomach, it was omitted; otherwise the treatment was continued.

*July 2d.*—Œdema of the extremities and surface has entirely disappeared; the effusion in the pleural and peritoneal cavities very much diminished; has had no more symptoms of asthma; complains only of weakness, and bids fair to make a rapid recovery. E. P. I.]

